# Expression profiling of disease progression in canine model of Duchenne muscular dystrophy

**DOI:** 10.1371/journal.pone.0194485

**Published:** 2018-03-19

**Authors:** Candice Brinkmeyer-Langford, Candice Chu, Cynthia Balog-Alvarez, Xue Yu, James J. Cai, Mary Nabity, Joe N. Kornegay

**Affiliations:** 1 Department of Veterinary Integrative Biosciences, Texas A&M University, College Station, TX, United States of America; 2 Department of Veterinary Pathobiology, Texas A&M University, College Station, TX, United States of America; University of Minnesota Medical Center, UNITED STATES

## Abstract

Duchenne muscular dystrophy (DMD) causes progressive disability in 1 of every 5,000 boys due to the lack of functional dystrophin protein. Despite much advancement in knowledge about DMD disease presentation and progression—attributable in part to studies using mouse and canine models of the disease–current DMD treatments are not equally effective in all patients. There remains, therefore, a need for translational animal models in which novel treatment targets can be identified and evaluated. Golden Retriever muscular dystrophy (GRMD) is a phenotypically and genetically homologous animal model of DMD. As with DMD, speed of disease progression in GRMD varies substantially. However, unlike DMD, all GRMD dogs possess the same causal mutation; therefore genetic modifiers of phenotypic variation are relatively easier to identify. Furthermore, the GRMD dogs used in this study reside within the same colony, reducing the confounding effects of environment on phenotypic variation. To detect modifiers of disease progression, we developed gene expression profiles using RNA sequencing for 9 dogs: 6 GRMD dogs (3 with faster-progressing and 3 with slower-progressing disease, based on quantitative, objective biomarkers) and 3 control dogs from the same colony. All dogs were evaluated at 2 time points: early disease onset (3 months of age) and the point at which GRMD stabilizes (6 months of age) using quantitative, objective biomarkers identified as robust against the effects of relatedness/inbreeding. Across all comparisons, the most differentially expressed genes fell into 3 categories: myogenesis/muscle regeneration, metabolism, and inflammation. Our findings are largely in concordance with DMD and mouse model studies, reinforcing the utility of GRMD as a translational model. Novel findings include the strong up-regulation of chitinase 3-like 1 (CHI3L1) in faster-progressing GRMD dogs, suggesting previously unexplored mechanisms underlie progression speed in GRMD and DMD. In summary, our findings support the utility of RNA sequencing for evaluating potential biomarkers of GRMD progression speed, and are valuable for identifying new avenues of exploration in DMD research.

## Introduction

Duchenne muscular dystrophy (DMD) is a devastating X-linked degenerative muscle disease affecting approximately 1 in 5,000 boys, caused by lack of functional dystrophin protein, Dystrophin is essential to muscle integrity via its role as a shock absorber that stabilizes the plasma membrane, or sarcolemma, of muscle cells against mechanical stress [1]. In the absence of dystrophin, myofibers undergo repeated cycles of necrosis and regeneration. As the disease progresses, stem cell reserves are depleted and muscle is replaced by fibrous connective tissue and fat.

Animal models of DMD have been essential to understanding the disease process and developing treatments. The well-known *mdx* mouse model has been used extensively to study disease pathogenesis and establish initial proof of principle for novel therapies [2]. The *mdx* model recapitulates the inflammatory environment seen in DMD-affected skeletal muscles and has been extensively used to examine the roles of inflammation in DMD [3]. However, while *mdx* mice lack dystrophin, they have a much less severe phenotype than DMD patients, raising questions about the degree to which preclinical data will translate to humans. Canine models of DMD, such as golden retriever muscular dystrophy (GRMD), more closely approximate phenotypic features observed in DMD [4]. In both DMD and GRMD, disease severity and progression differ among individuals and muscles. Phenotypic variation in dystrophin-deficient species, individuals, and muscles suggests that modifier genes can influence the disease course. Gene expression array studies in the *mdx* mouse [e.g., 3, 5], GRMD dogs [6, 7], and DMD patients [8–10] have allowed identification of key potential genetic modifiers. These studies have shown that genes tied to inflammation and regeneration are particularly increased in dystrophic muscle, presumably in response to myofiber necrosis.

Genes that influence the speed of disease progression in dystrophin deficient individuals are of particular interest because they could potentially be therapeutic targets. In this study, we compared gene expression profiles of two groups of differentially affected GRMD dogs at 3–6 months of age, a period of particularly fast disease progression that corresponds to the 5–10 year period in DMD [11]. In keeping with prior gene expression studies, we hypothesized that dogs with more rapid disease progression would have a corresponding increase in genes associated with inflammation and regeneration and that differential expression would be particularly pronounced at the 3-month time point, as a prelude to fibrosis.

This study is one of the first examples of the use of RNA sequencing being used as a tool to investigate contributory genetic elements in a canine model of disease. It is also the first study to use RNA sequencing to investigate disease progression in GRMD dogs (though it should be noted that whole genome DNA sequencing has been done previously; for example [12, 13]). Because phenotypically characterized samples were used, we anticipate that our findings will be relevant to DMD studies exploring analogous phenotypes in DMD.

## Methods

### Animals

All GRMD dogs were from the colony currently located at Texas A&M University, but located at the time of biopsy/necropsy at University of North Carolina-Chapel Hill (UNC-CH). This colony has been maintained through a breeding program in which at least one parent of each mating carries the same causal mutation inherited from a single founding sire [14]. Once the inbreeding coefficient of the colony reaches ~0.20, unrelated dogs are introduced to the breeding stock to reduce neonatal mortality (which increases with inbreeding) [4]. As a result of this practice, variation is observable in the progression of disease and severity of the phenotype [15], giving the colony many of the advantages of an inbred population (e.g. reduced influence of confounding factors such as environmental variation), while capturing genetic diversity such as that of humans. As a result, studies using the GRMD colony require a smaller sample size than would a study using humans [16].

**Fig 1:**
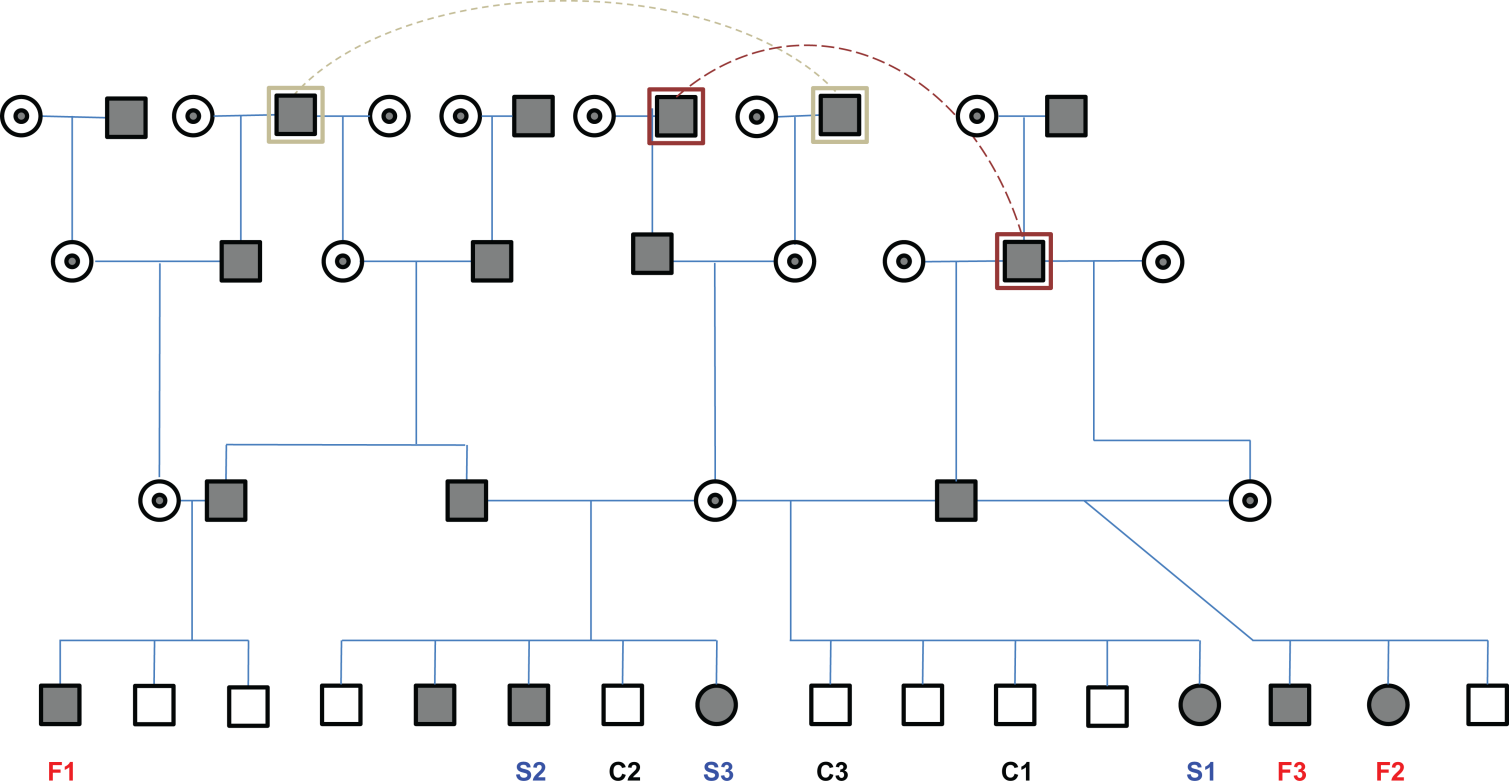
Pedigree of dogs used in this study. Note that this pedigree does not show the entire colony or all siblings of the dogs used in this study, but is presented to illustrate the degree of relatedness between dogs in this study. Circles represent females; squares represent males; open symbols represent non-GRMD (non-dystrophic); blackened symbols represent GRMD (dystrophic); a small circle within a larger circle represents GRMD carrier females; animals listed in more than one position within the pedigree are indicated by a larger circle or square encompassing the primary symbol for the animal, along with a dotted line connecting the multiple positions for that animal in the pedigree. The 9 dogs investigated for this study are listed across the bottom of the pedigree, with “fast” and “slow” labels indicating disease progression speed for GRMD dogs and “control” indicating non-GRMD dogs.

The dogs were maintained and treated according to the standards of the National Research Council Guide for the Care and Use of Laboratory Animals. Studies were approved by the UNC-CH Institutional Animal Care and Use Committee (IACUC) through protocols UNC IACUC 09–351, Standard Operating Procedures—Canine X-Linked Muscular Dystrophy. The GRMD genotype was diagnosed based on blood creatine kinase levels taken shortly after birth [17], and confirmed with PCR-based genotyping [18]. Nine dogs were included in this study based on the availability of longitudinal samples and phenotypic data. This cohort included 6 GRMD-affected and 3 unaffected dogs with samples taken via biopsy at ~3 months (time point 1, T1) and via biopsy or necropsy at ~6 months (time point 2, T2) of age. Of the affected dogs, 3 were classified as have slow-progressing disease and 3 were classified as fast-progressing, based on measurement values for objective GRMD biomarkers [4] found to be reliable indicators of GRMD disease severity. These biomarkers included tibiotarsal joint tetanic extension (N/kg), percent eccentric contraction decrement, maximum hip flexion angle, and pelvic angle [19]. We previously identified these particular biomarkers as having genomic inflation factors (λ) of 1, meaning that measurements for these biomarkers are useful for genetic association studies because their values are independent and not biased by pedigree relationships [20]. Each of the GRMD subsets consisted of 2 affected females and 1 affected male. The unaffected dogs were all males. A pedigree of the 9 dogs is presented in Fig 1. We studied the cranial sartorius (CS), a flexor muscle which is more severely affected than extensor muscles (such as the vastus lateralis) and undergoes a characteristic pattern of early necrosis with hypertrophy by 6 months [7, 21]. CS hypertrophy, reflecting GRMD disease severity, has been previously correlated with measurement values for the aforementioned biomarkers [7].

### RNA extraction

Total RNA was extracted from biopsy and necropsy samples from CS muscles archived at -80^o^ C for each of the 9 dogs using TriPure Isolation Reagent (Roche; Indianapolis, IN) as per manufacturer’s instructions. RNA was precipitated using isopropanol and 75% EtOH, and dissolved in nuclease-free water (Ambion). To minimize DNA contamination, samples were DNase-treated using the DNA-Free DNase Treatment and Removal kit (Ambion). RNA was quantified using NanoDrop (Thermo Fisher Scientific) and quality was evaluated via Bioanalyzer (Agilent; RIN values ranged from 8 to >9).

### Real-time quantitative PCR (RT-qPCR)

Total RNA was directly reverse transcribed to cDNA using SuperScript II (Invitrogen). DNase-treated RNA (100ng) was reverse transcribed with oligo-dT and random primers and Superscript II (Invitrogen, Carlsbad, CA). The reverse transcription reactions consisted of 100ng of DNase-treated RNA in a 50μl reaction, ultra pure water, oligo dT (2.5μl of 500ng/μl) and random hexamer (0.48μl of 1mM stock) heated to 65°C for 5 minutes and cooled to room temperature. To this, Superscript II (2μl), 5X 1st Strand buffer (10μl), 0.1M DTT (5μl), 10mM dNTPs (2.5μl) and an RNase block Ambion’s Superasin (1μl) were added. All were heated to 37°C for one hour followed by reverse transcription inactivation at 90°C for 5 minutes.

SYBR Green technology was used to measure expression levels for CHI3LI, IL-6, SPP1, and the housekeeping gene hPRT. Primers were designed from two neighboring exons flanking one intron (when possible), or from a single exon, using Primer3 software (http://biotools.umassmed.edu/bioapps/primer3_www.cgi; [22]). Each 20μl reaction contained 10μl Power SYBR Green PCR Master Mix (Applied Biosystems), 2μl each forward and reverse primers (3μM each), 5.5μl ultra-pure water, and 0.5μl (1ng) cDNA. All RT-qPCR reactions were performed in triplicate using the 7900HT Fast qPCR System (Applied Biosystems); cycling parameters were 50°C for 2 minutes, 95°C for 10 minutes, and cycling 40 repeats of 95°C for 15 seconds and 60°C for 1 minute.

Primer information is listed in Table 1. Relative expression data were calculated using the ΔΔCt method of Livak and Schmittgen [23], and normalized to the housekeeping gene hPRT. Differential gene expression was statistically evaluated via ANOVA using JMP version 13 software [24]. In all cases, *p*≤0.05 was considered significant.

**Table 1:** RT-qPCR target and reference gene primers for RNAseq validation.

GENE	RefSeq	F primer (5’-3’)	R primer (5’-3’)
CHI3L1	NM_001177807.1	AGACCCTCCTGTCTGTTGGA	ACTCTGGGTCTTGGAGGCTA
HPRT	NM_001003357.2	AGCTTGCTGGTGAAAAGGAC	TTATAGTCAAGGGCATATCC
IL-6	NM_001003301.1	CTCGGCAAAATCTCTGCACTG	TGTGAAGACAGCAAAGAGGCA
SPP1	XM_003434023.4	GGGAGCTCTGAGGAAAAGCA	GCTTCTGAGATGGGTCAGGC

### RNA sequencing

Coding transcriptome sequence was captured using TruSeq RNA library preparation kits (Illumina, catalog numbers RS-122-2001 and RS-122-2002) and rRNA removed using RiboZero Gold (Illumina). TruSeq-barcoded RNA sequencing libraries were analyzed using the Illumina HiSeq 2500v4 High Output Genome Analyzer. Read lengths were 125bp (paired-end), with 220-225M reads per lane. The resulting sequence was evaluated for quality using FastQC (version 0.11.2). The dog genome sequence and assembly files (CanFam3.1) were used for mapping and alignments by HISAT2 (version 2.0.3-beta) [25]. Raw read counts and alignments were performed using SAMtools (version 1.2) and the “union” mode of HTseq (version 0.6.1) [26]. High performance computing resources at Texas A&M University (http://hprc.tamu.edu) were used for HISAT2 and HTseq analyses.

The datasets generated during and/or analyzed during the current study are available from the corresponding author on reasonable request.

### Statistical analysis

The R (version 3.2.4)-based Bioconductor (version 3.3) package DESeq2 (release 3.3) [27] was used for statistical analyses. Un-normalized raw reads were used as input, because DESeq2 corrects internally for library size; we also took into consideration differences in sequencing depth by using variance stabilizing transformation [27]. The Benjamini-Hochberg approach was used to adjust P-values for multiple testing [28]. For the selection of differentially expressed (DE) genes, we used a false discovery rate (FDR) adjusted p-value (i.e., q-value) of < 0.05.

### Gene ontology and pathway analysis

The PANTHER Overrepresentation Test in PANTHER version 11.0 [29, 30] was used to perform gene ontology and PANTHER pathways analyses. Furthermore, we used Ingenuity Pathway Analysis (IPA, QIAGEN Redwood City, www.qiagen.com/ingenuity) to identify orthologous genes and pathways in human, mouse, and rat databases. Multiple testing was performed in PANTHER suing the binomial test and Bonferroni correction, and in IPA using the right-tailed Fisher Exact test. For both PANTHER and IPA, we used default settings for statistical analysis, with p-value < 0.05 and log_2_ fold change >2 set as cutoff values.

### Single nucleotide variant (SNV) discovery and functional assessment

We pooled short reads generated from RNAseq of dogs affected with disease (including both slow and fast progression groups) as one group and the healthy dogs as another group. The resulting read files were aligned to the dog (*Canis lupus familiaris*) reference genome using the STAR 2-pass alignment [31]. Picard (http://broadinstitute.github.io/picard) was used to add read group information, sorting, marking duplicates and indexing. We then used a function of the GATK tool called SplitNCigarReads to split reads into exon segments to get rid of *N*s but maintain grouping information, and hard-clip sequences overhanging into the intronic regions. The variant calling was performed by using another GATK function—HaplotypeCaller. To filter the resulting callset, hard filters (FS > 30.0, QD < 2.0 and DP > = 20, where FS stands for Fisher Strand values, QD for Quality by Depth and DP for Depth of Coverage) were applied ([32–34],https://software.broadinstitute.org/gatk/documentation/article.php?id=3891). After variant calling for diseased and control dogs independently, we compared the two groups to obtain the list of SNVs present only in the GRMD dogs. We predicted the effect of each SNV on protein function using the Variant Effector Predictor at the Ensemble website (https://useast.ensembl.org/info/docs/tools/vep/index.html).

The code used is available at https://github.com/XueMaryYu/Dog_variant_calling.

## Results

To evaluate the relationship between gene expression and speed of GRMD disease progression, we performed RNA sequencing analysis using cranial sartorius muscle from 9 dogs classified as GRMD slow-progression (*n* = 3), GRMD fast-progressing (*n* = 3), or unaffected dogs from the same colony (*n* = 3). After removal of low-quality reads (Phred score >30) a total of over 400 million reads (average of 24 million per dog) were mapped to the canine genome, CanFam3.1, with alignments ranging from 95.09–96.38%. Of these alignments, 76.25–80.84% were uniquely mapped. See Table 2 for a summary of RNA sequencing results.

**Table 2:** Summary of RNA sequencing results.

	Condition	Dog Names	Input Paired-end Reads	Overall Alignment rate (%)	Uniquely mapped Paired-end reads (%)	.gz File size(G)	Number of genes in raw counts
T1	**Fast**	F1	19342545	96.19	80.84	3.0	16868
	**Fast**	F2	23347374	95.79	79.24	3.4	16861
	**Fast**	F3	14777876	95.62	76.25	2.4	16126
	**Slow**	S1	22893262	96.27	79.47	3.4	16709
	**Slow**	S2	30852701	95.84	79.03	4.6	17372
	**Slow**	S3	47965909	95.82	78.85	7.1	17770
	**Control**	C1	14195971	95.09	76.3	2.2	15343
	**Control**	C2	15993887	95.96	77.24	2.5	15730
	**Control**	C3	23913980	95.87	77.02	3.6	16230
T2	**Fast**	F1	29086802	95.99	79.37	4.5	17234
	**Fast**	F2	39677312	95.9	79.15	6.0	17575
	**Fast**	F3	17497417	96.13	78.55	2.7	16322
	**Slow**	S1	27696607	95.99	78.99	4.1	16673
	**Slow**	S2	10908787	95.7	79.54	1.6	15569
	**Slow**	S3	19656259	96.18	79.73	3.0	16583
	**Control**	C1	26523841	96.15	76.7	3.8	15637
	**Control**	C3	22436687	96.38	77.6	3.4	15818

### Principal component analysis (PCA) and hierarchical clustering

**Fig 2:**
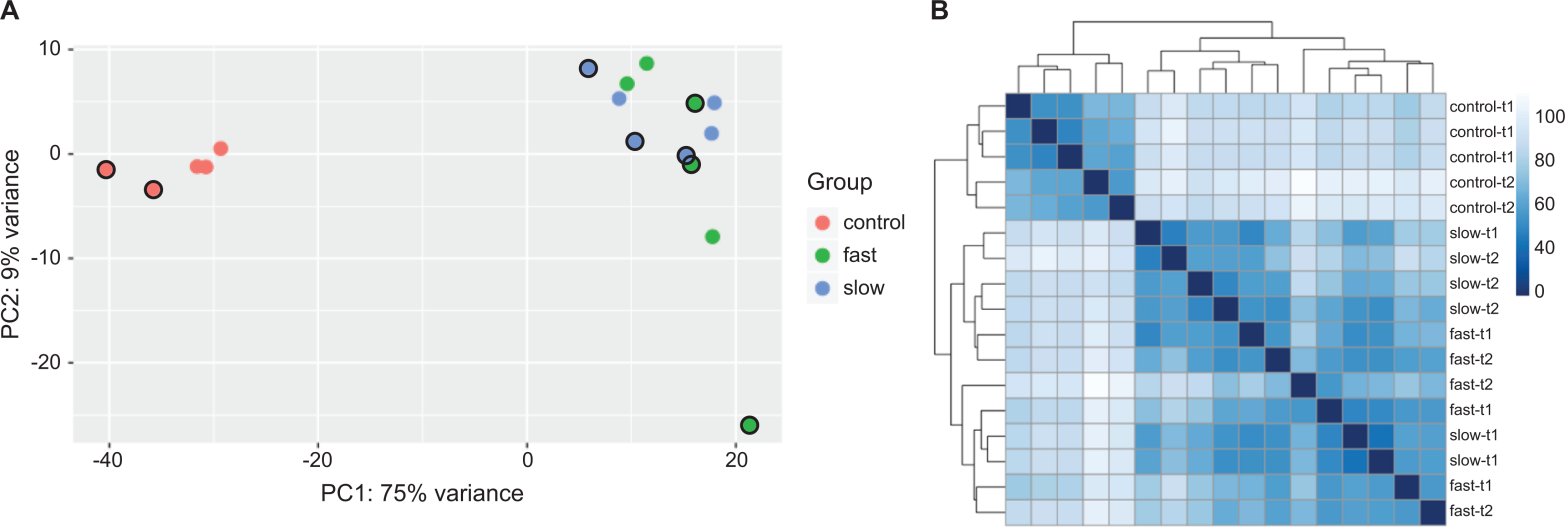
Principal component and hierarchical analysis for all dogs at both time points. Principal component 1 (PC1) and Principal component 2 (PC2) were identified by logarithm transformation in DESeq2 at two time points. 75% and 9% variance were explained by PC1 and PC2, respectively. A) shows the principal component analysis for the three groups of dogs: red circles indicate controls, green represents fast-progressing dogs, and blue represents slower-progressing dogs. B) shows the principal component analysis for the two time points: here, red circles represent T1 (age 3 months), and green circles represent T2 (age 6 months). C) is a heatmap showing sample-to-sample distances. Distance was analyzed by logarithm transformation in DESeq2.

Using DESeq2, we performed principal component and hierarchical clustering analyses to identify underlying stratification across samples (Fig 2). All control dogs clustered separately from GRMD samples for both types of analysis. GRMD dogs did not show additional clustering when viewed by time point or progression speed.

### Differentially expressed genes between groups

**Fig 3:**
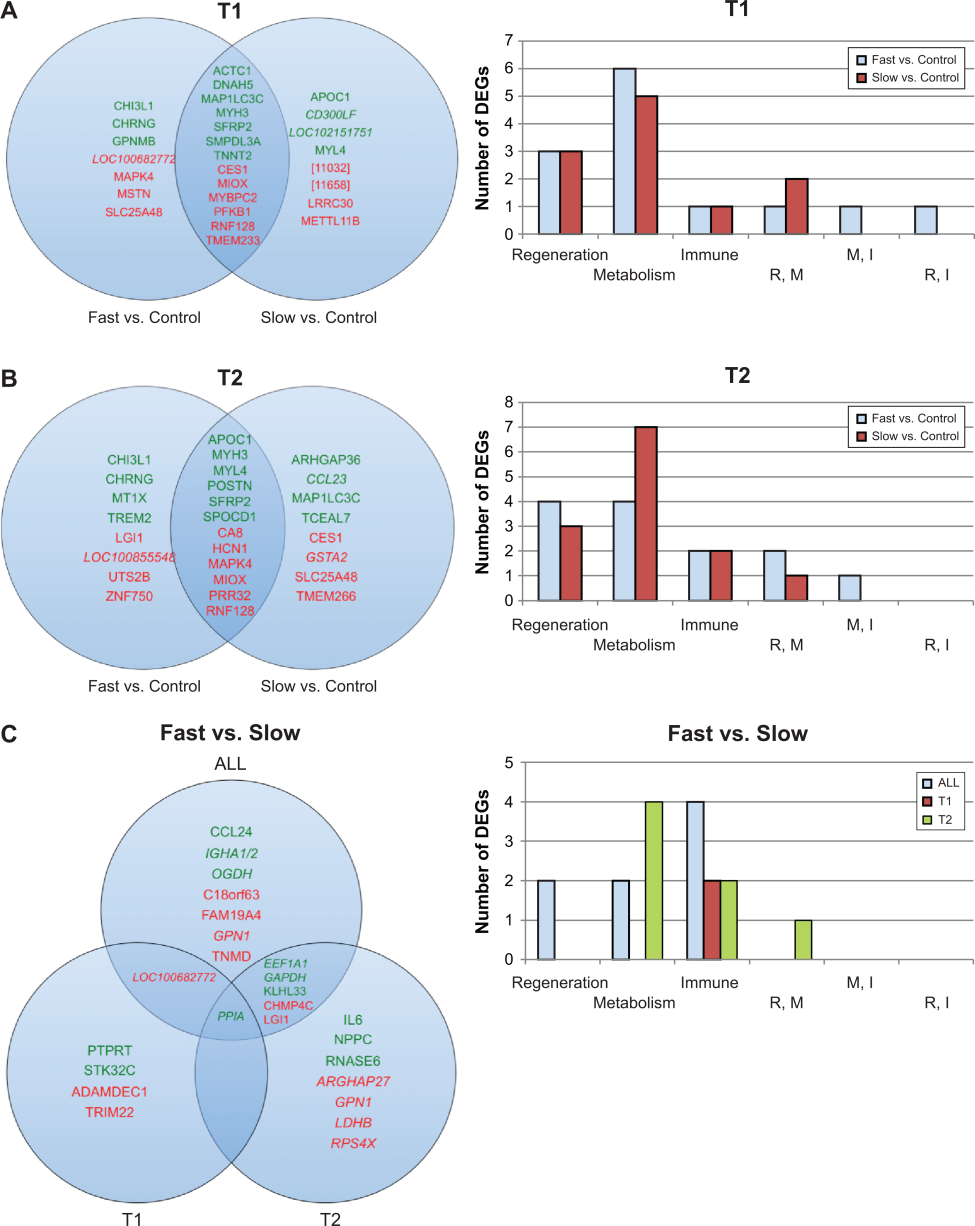
Top 10 DEGs for A) T1: age 3 months, GRMD vs. control; B) T2: age 6 months, GRMD vs. control; C) fast vs. slow progressing GRMD dogs. Green font indicates up-regulated genes; those genes in red font were down-regulated. Venn diagrams on the left of each panel include names of Top 10 DEGs; graphs on the right of each panel indicate numbers of DEGs with functions related to regeneration, metabolism, immune response, both regeneration and metabolism (R,M), both metabolism and immune response (M,I), or both regeneration and immune response (R,I).

We evaluated gene expression differences between dogs with fast- vs. slow-progressing GRMD disease, both GRMD groups vs. unaffected controls, and gene expression at 3 and 6 months representing early (T1) and stable (T2) disease stages, respectively (Fig 3). Using DESeq2, we identified those genes with significant differential gene expression (considered as q < 0.05; Table 3). Similar to human DMD and *mdx* mouse studies, we found differences in gene expression were greatest in those genes with functions pertaining to inflammation/immune response, metabolism, and myogenesis/muscle regeneration. Furthermore, these processes are often interrelated, with several of the most strongly differentially expressed genes (DEGs) having roles in both inflammation and regeneration. Top 10 DEGs, log_2_ fold changes, and q-values are listed in S1 Table.

**Table 3:** Numbers of differentially expressed genes (DEGs) and gene ontology analysis output. Total numbers of DEGs for the comparisons “Fast vs Control”, “Slow vs Control”, and “Fast vs Slow” are presented in gray rows, with these results broken down by time points below the total numbers (T1 = age 3 months; T2 = age 6 months). For DESeq2, genes with q value < 0.05 were considered as significant. For Gene Ontology, Kyoto Encyclopedia of Genes and Genomes (KEGG), and Panther, genes matched with *Canis familaris* genome in respective database with q value<0.05 were reported.

Samples	DESeq2			Gene ontology				Panther
	Total	Up	Down	Mapped genes	Molecular function	Biological process	Cellular component	
**Fast vs Control**	6503	3539	2964	6156	7	13	3	2
**T1**	3789	2108	1681	3593	9	16	4	6
**T2**	4516	2517	1999	4256	9	15	5	2
**Slow vs Control**	7338	3790	3548	6979	5	7	3	1
**T1**	5141	2615	2526	4884	7	11	3	3
**T2**	4118	2158	1960	3922	9	13	7	2
**Fast vs Slow**	2459	1329	1130	2342	1	3	2	0
**T1**	6	3	3	6	0	0	0	0
**T2**	2275	1139	1136	2133	2	2	2	0

### DEGs between GRMD and unaffected control dogs

#### a. DEGs related to muscle regeneration

Based on findings from studies using *mdx* mice [35–37] and DMD patients [8–10], we hypothesized that there would be an increase in expression of genes related to muscle regeneration in GRMD compared to control dogs. Myosin genes are expressed at different stages of muscle development, subsequently down regulated at birth, and then re-expressed during muscle regeneration [38]. Consistent with our hypothesis, expression of the embryonic form of myosin heavy chain (MYH3), associated with muscle regeneration following injury, was increased ~7-fold at both time points in all GRMD dogs compared to controls, suggesting that muscle regeneration is a hallmark of GRMD for at least the first 6 months. MYH3 has previously been shown to be over-expressed in DMD [10] and has also been correlated with muscle regeneration in *mdx* mice [35]. Myosin light chain 4 (MYL4) is expressed later than MYH3 during normal mouse embryogenesis, corresponding to the beginning of the fetal stage of muscle development and the time of fiber type differentiation [38, 39]. Not surprisingly, we found MYL4 to be among the most up-regulated genes in all GRMD dogs at the later (6-month) time point. Similarly, increased expression of cardiac troponin type 2 (TNNT2) has been described in transcriptome analyses of DMD and *mdx* [40, 41]; this up-regulation coincides with early myoblast activation followed by a gradual decline in expression corresponding with maturation of the regenerated muscle [40]. TNNT2 is strongly expressed at later stages in embryonic skeletal muscle [42]. In keeping with this pattern, TNNT2 was one of the most up-regulated genes in all GRMD dogs at the earlier (3-month) time point.

#### b. DEGs related to metabolism

In GRMD and DMD (and, to a lesser extent, *mdx*), skeletal muscles show aberrations in energy metabolism, or “metabolic crisis.” Based on prior studies [43, 44], we anticipated decreased expression for genes involved in energy metabolism. In fact, many of the top 10 DEGs that were down-regulated in GRMD relative to control–regardless of time point or progression speed–had functions related to metabolism. Myo-inositol oxygenase (MIOX) was down-regulated by as much as 5-fold in all GRMD dogs at both time points. Carboxylesterase 1 (CES1) was down-regulated by ~4-fold in slow-progressing dogs at 3 months and all dogs at 6 months. Solute carrier family 25 member 48 (SLC25A48; encoding a mitochondrial carrier protein) was down-regulated in fast-progressing dogs at the 3 month time point and in slow-progressing dogs at the 6 month time point (>3-fold in all GRMD dogs).

#### c. DEGs related to inflammation

Inflammation plays a major role in disease progression in DMD and *mdx*. Genes with roles in inflammatory and immune-related processes were included among the top 10 most strongly up- or down-regulated genes in all comparisons. Of these genes, chitinase 3-like 1 (CHI3L1) is particularly worth noting. CHI3L1 was found to be up-regulated ~6-fold in fast-progressing dogs compared to controls at both time points.

### DEGs between fast- and slow-progressing GRMD dogs

One of the main purposes for this study was to identify differences in gene expression between fast- and slow-progressing GRMD dogs that could help define what causes some dogs to deteriorate faster than others, thus giving insight into potential avenues for investigation in DMD. At T1, the most up-regulated DEG in fast-progressing compared to slow-progressing GRMD dogs was protein tyrosine phosphatase, receptor type T (PTPRT). This gene encodes a signaling molecule that regulates several processes including cell growth and differentiation. In humans, PTPRT has been associated with autism [45–47], which is comorbid with DMD [48]. On the other hand, the most strongly down-regulated gene at T1 when comparing fast-progressing to slow-progressing GRMD dogs was ENSCAFG00000031467, also called LOC100682772 leucine-rich repeat-containing protein 37A3-like. This gene was also the most down-regulated gene overall in fast- vs. slow-progressing dogs. The protein encoded by this gene is a member of the leucine-rich repeat containing family, the members of which have been shown to regulate collagen fibrillogenesis [49].

At T2, the most highly up-regulated gene in fast-progressing (as compared to slow-progressing) GRMD dogs was interleukin 6 (IL-6). IL-6 is affiliated with the innate immune response and its increased expression in fast vs. slow-progressing dogs likely reflects a stronger inflammatory reaction due to a relatively larger number of regenerative muscle fibers. The most down-regulated gene in fast-progressing dogs relative to slow-progressing dogs was a novel processed pseudogene (ENSCAFG00000032111) with no known orthologs or relevant literature to provide clues as to its function. In fact, many of the other top-hit DEGs in the fast vs. slow-progressing GRMD comparison were novel genes and pseudogenes for which little functional information was available.

Only one gene was found to be among the top 10 DEGs at both time points when comparing expression levels between fast- and slow-progressing dogs. This gene, ENSCAFG00000014968, is an ortholog of peptidylprolyl isomerase a (PPIA) and was previously identified as a gene of interest in our genome-wide association study [19]. We had originally considered PPIA as a potential housekeeping gene for normalization in that previous study, but instead in that study we observed PPIA expression to be significantly different in 6- and 12-month-old GRMD compared to normal dogs in the cranial tibialis muscle. PPIA is a regulator of a several processes relevant to GRMD, including inflammatory response to injury and apoptosis in certain cell types such as smooth muscle and endothelial cells [50, 51].

### Gene ontology and pathway analyses for DEGs related to speed of disease progression

Results for gene ontology and pathway analysis for comparisons across fast, slow, and control dogs are found in S2 Table.

#### Inflammation

As expected, gene expression pathways related to inflammation were enriched in GRMD dogs compared to control dogs at both time points and progression speeds. At 3 months of age, significantly up-regulated genes in the fast-progressing GRMD dogs included those involved in T-cell activation. This suggests a strong early immune response in these dogs, likely in reaction to damage-associated molecular patterns, as seen in *mdx* mice [52]. Migration of regulatory T cells to damaged muscle can ameliorate the degenerative phenotype, allowing for muscle repair early in the disease process [53, 54]; however, T-cell activation was not identified as a top pathway for these dogs at age 6 months. This would be in keeping with the disease course of the CS in GRMD, with necrosis occurring even in utero and then stabilizing [7, 21]. Interestingly, T-cell activation was not a top pathway for slow-progressing dogs at either time point.

#### Growth and regeneration

Angiogenesis and cholecystokinin (CCK) signaling were among the top most-enriched pathways in all GRMD dogs at age 3 months, consistent with a regenerative phenotype at this younger age together with changes to neuromuscular signaling. The integrin signaling pathway was enriched in all GRMD dogs at 6 months of age, consistent with our prior studies [55], perhaps as part of a compensatory mechanism to ameliorate increasing amounts of muscle damage [56, 57].

At age 3 months, the fast-progressing GRMD dogs exhibited higher levels of expression in genes involved in the platelet-derived growth factor (PDGF) signaling pathway and tricarboxylic acide cycle pathway [18]. These two pathways were not as enriched in comparisons of control dogs vs. fast-progressing dogs at 6 months of age, or slow-progressing dogs at either time point; therefore, these pathways may also be critical to the more rapid progression of GRMD disease. This is not surprising, given the functions of these pathways: PDGF signaling is involved in growth, particularly blood vessel formation; while the TCA cycle is generally related to energy and metabolism.

### RT-qPCR of specific genes of interest

**Fig 4:**
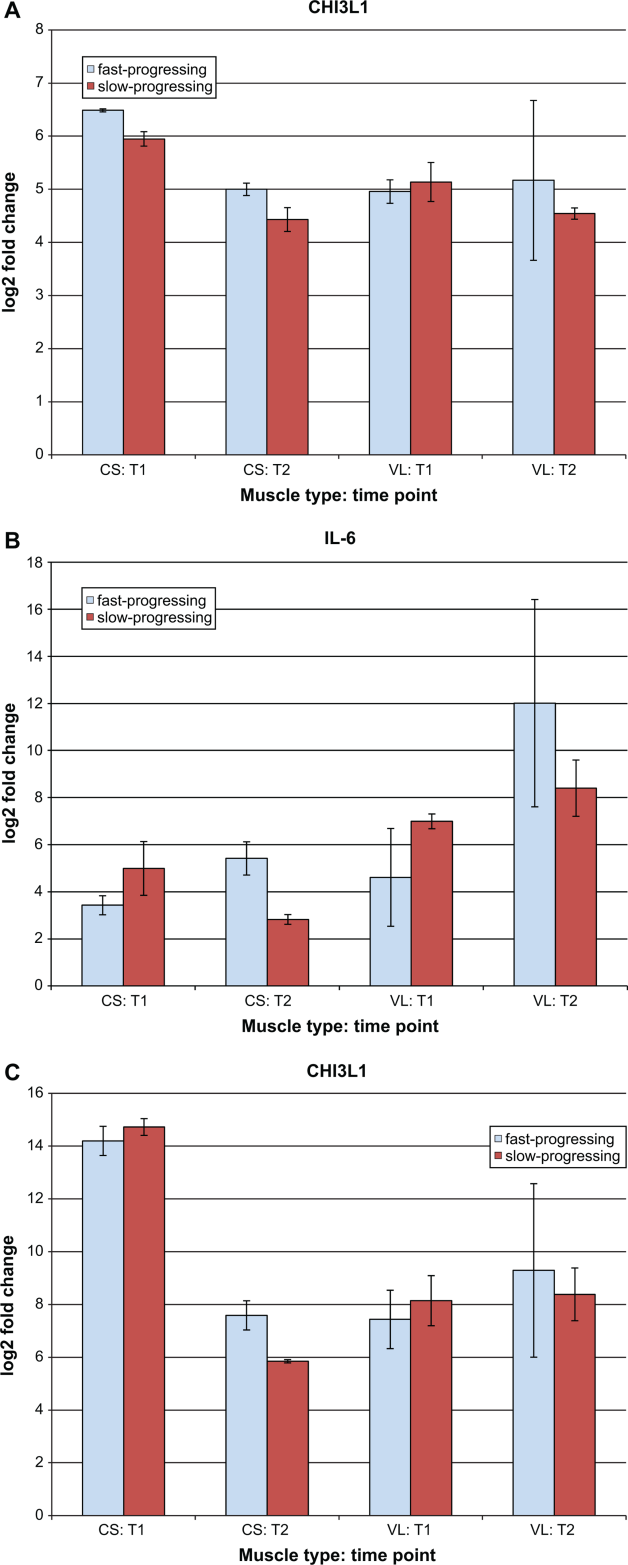
RT-qPCR confirmation of RNAseq results for selected genes at both time points evaluated, and two muscle types, in both fast- and slow-progressing dogs. The y-axes show the log2 fold change values, while the x-axes provide information about the samples (muscle type and time point). CS = cranial sartorius; VL = vastus lateralis; T1 = age 3 months; T2 = age 6 months. All are normalized to unaffected control dogs and to hPRT using the ΔΔCt method [23]. Error bars show standard error of means.

Expression profiles for CHI3L1, IL-6, and SPP1 were further evaluated using RT-qPCR (Fig 4). These genes were selected based on their association with GRMD progression speeds, especially in conjunction with processes (such as inflammation) likely to be affiliated with fibrosis. We chose to measure expression in the same tissue used for RNAseq (CS) as well as in the vastus lateralis (VL), an extensor muscle which functions in the opposite direction as CS (which is a flexor) [58, 59]. While no statistically significant differences were identified between expression levels of any of these genes in fast and slow progressing dogs, age had a significant effect on expression levels for CHI3L1 and SPP1. Muscle type also had a significant effect on SPP1 expression levels.

Comparisons of CHI3L1 expression levels were mostly in agreement between RNAseq and RT-qPCR: as observed via RNAseq, RT-qPCR results showed CHI3L1 expression was approximately 6 times higher in fast-progressing GRMD dogs than controls at T1. However, slow-progressing dogs also showed a nearly 6-fold increase in expression at T1, and both fast- and slow-progressing dogs showed an increase of nearly 5-fold at T2, based on RT-qPCR (but not RNAseq) results. The expression of IL-6 in the CS at T2 follows the same overall pattern in both RNAseq and RT-qPCR, in that IL-6 was up-regulated in fast-progressing GRMD dogs compared to slow-progressors, though RNAseq data showed this difference to be greater (2.59 fold via RT-qPCR, vs 3.97 fold via RNAseq). SPP1 expression in the CS was up-regulated in slow-progressing GRMD dogs compared to controls for both RT-qPCR and RNAseq.

Somewhat surprisingly, expression patterns were similar in VL compared to CS for all 3 genes–in other words, genes higher expression levels in the CS of fast- vs slow-progressing GRMD dogs also had higher expression levels in the VL for fast vs slow. The only exception here was for CHI3L1 at T1 (fast-progressing GRMD dogs had relatively higher fold change levels at T1 in the CS, but not VL, as compared to slow-progressing dogs). Given the opposing nature of CS and VL functions, the expression pattern similarity almost certainly reflects a broader pattern of expression changes related to progression speed rather than the influence of muscle type. In the cases of the 3 genes evaluated here, this “broader pattern” could reflect an overall increase in inflammatory markers in fast-progressing vs slow-progressing GRMD dogs. However, due to small sample sizes and large standard error values, expression comparisons in the VL—while intriguing—will require larger sample sizes before conclusions can be drawn.

### GRMD-associated SNVs and their potential impacts

**Fig 5:**
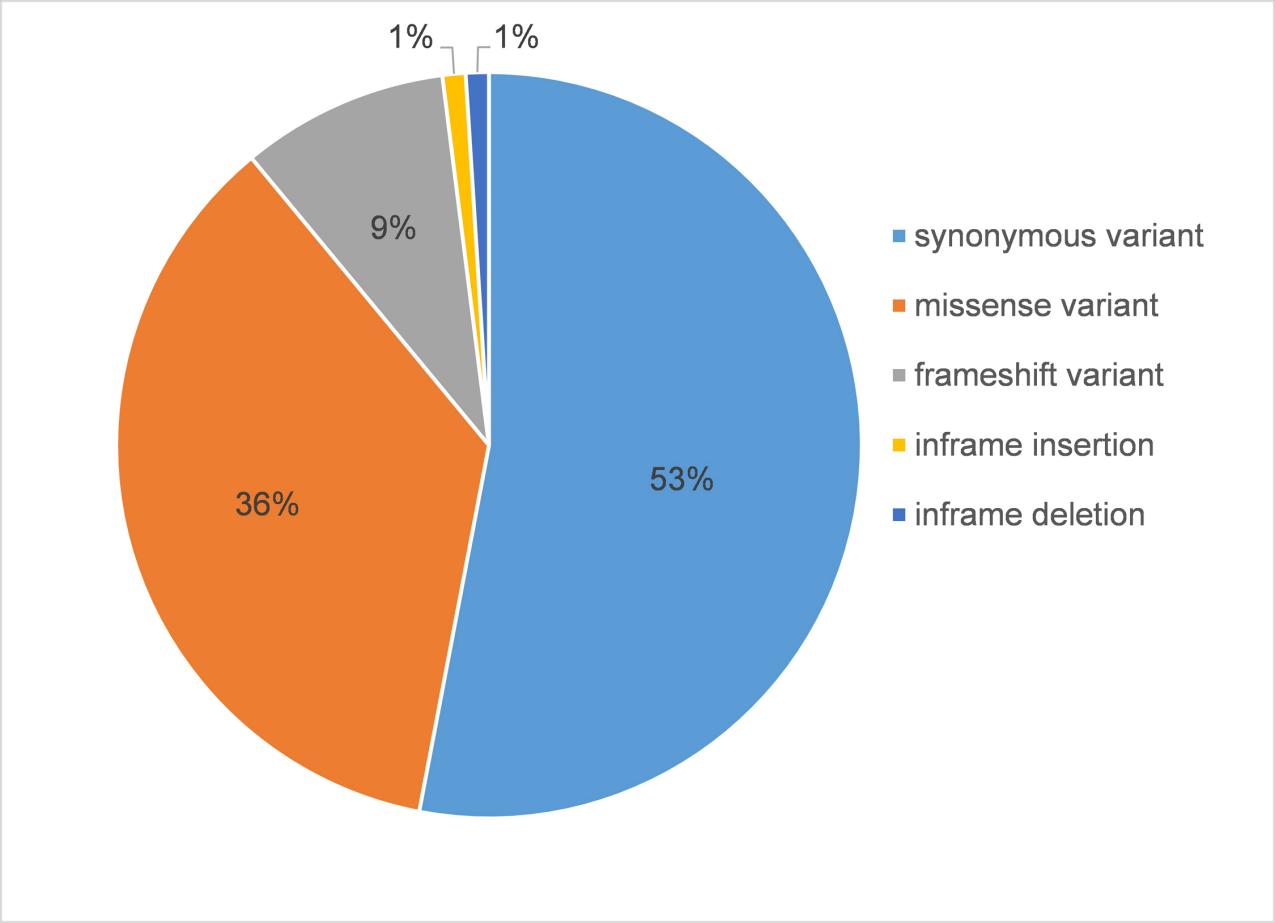
Classifications of coding SNVs found in GRMD dogs (but not normal dogs). Each SNV class is represented by a different color, with relative proportions of each shown as a pie chart.

Next, we set out to use the mapped RNA-seq reads to identify SNVs that are present in the genomes of dogs affected with GRMD but absent in those of unaffected dogs. These identified SNVs should be located in the transcribed regions and some of these SNVs located in coding regions are likely to alter the function of proteins. Using a GATK-based SNV identification pipeline (Materials and Methods), we identified 13,108 GRMD-specific coding SNVs (with minimal read depth = 20). These coding SNVs are composed of 53% synonymous variants, 36% missense variants, 9% frameshift variants, 1% in-frame insertions, and 1% in-frame deletions (Fig 5). A total of 6,066 missense and frameshift SNVs were predicted to have a moderate or high impact on protein function (S3 Table). Altogether, there are 2,693 proteins that contain at least one of these GRMD-specific SNVs.

Of the GRMD-specific coding SNVs, 32 SNVs are located within the 16 of the top 10 up- or down-regulated DEGs in any given comparison (such as fast vs. slow progressing GRMD, or GRMD vs. control, at T1, T2, or overall). In 8 cases, multiple SNVs are present within the same gene: ADAMDEC1, ARHGAP36, ENSCAFG00000024944, ENSCAFG00000032099, ENSCAFG00000032358, F5, SMPDL3A, and TRIM22. There are no obvious effects of these SNVs on GRMD phenotype, but future studies are warranted, as the affected genes have roles in metabolism as well as immune response and could potentially influence disease progression.

## Discussion

We have evaluated gene expression differences relevant to disease progression speed based on a series of quantitative, objective biomarkers [4]. Many of our findings from comparing GRMD to unaffected control dogs echo those of human and mouse studies, as well as prior array-based GRMD gene expression analyses [e.g., 6, 7]. This reaffirms GRMD as a relevant model for studying DMD. Furthermore, our findings provide evidence of genetic modifiers of GRMD, in addition to those found in our previous genome-wide association study [19].

Perhaps more interesting, however, are findings related specifically to speed of disease progression, which may open new avenues of exploration in the field of GRMD/DMD research. When we compared healthy dogs to GRMD dogs, we identified potential genetic biomarkers of disease progression. Most of these genes fall under one or more of three main categories: muscle regeneration and growth, metabolism, and inflammation.

To gain insight into how differences in muscle regeneration could affect disease progression, we took a closer look at the ontology of significant PANTHER pathways specifically in fast-progressing GRMD dogs early in the disease course–the 3-month time point. In those dogs, we found increased expression levels in genes included in the PDGF pathway, which is involved in growth and angiogenesis, and likely related to clearing of necrotic debris and regeneration of damaged muscle. PDGF staining has previously been associated with regenerative fibers in muscular dystrophies, including DMD and *mdx* [60, 61]. These studies showed that once the disease process advanced beyond the regenerative stage to fibrosis, PDGF was no longer observed in dystrophic muscles. In fact, PDGF signaling has been associated with mesenchymal progenitors involved in adipogenesis and fibrosis [62, 63]. PDGF signaling was not among the pathways enriched in 6-month-old GRMD dogs, regardless of disease progression speed. This suggests that by age 6 months the rapidly progressing GRMD dogs had reached a point where fibrosis had become more prominent than regeneration, in keeping with the acute onset of CS necrosis in GRMD and relative stability by 6 months. The fact that PDGF signaling was not among the enriched gene ontology pathways for slow-progressing GRMD dogs at either time point would be consistent with less pronounced early necrosis and reduced or slowed accumulation of muscle damage, adipogenesis and fibrosis. These findings provide further support to the idea that blocking PDGF signaling may ameliorate fibrosis in dystrophic muscles, a concept currently being explored as a potential treatment for DMD [64].

While genes with functions related to muscle regeneration were often up-regulated in GRMD dogs, many of the top DEGs related to metabolism were down-regulated in GRMD dogs compared to controls. Down-regulation of those DEGs could contribute to muscle degeneration or failed regeneration, aggravating the ongoing battle to compensate for disease-related muscle loss. Genes that inhibit myofiber differentiation and growth counter the muscle regenerative process. Indeed, genes that promote and inhibit muscle differentiation must be held in balance to avoid either atrophy or hypertrophy [65]. In recent years, there has been considerable interest in blocking inhibitory genes to promote greater muscle regeneration in muscular dystrophy and other muscle wasting disorders [66]. Myostatin (MSTN) is a member of the TGF-β family that acts as a negative regulator of muscle growth [67, 68]. Inhibition of MSTN signaling enhances muscle growth and regeneration in the mdx mouse [69–71] and both normal [72] and GRMD [73] dogs. The results of previous studies have shown that MSTN is naturally down-regulated by the body in muscular dystrophy, perhaps to promote muscle regeneration [5, 7, 9]; therefore, we hypothesized that we would find decreased expression of MSTN in the dystrophic dogs, more greatly reduced in the fast-progressing group and especially at the earlier time point (3 months). Indeed, we observed MSTN expression levels reduced over 3-fold in the fast-progressing GRMD dogs compared to control dogs at age 3 months. However, at 6 months there was little difference in MSTN expression between these two groups, and at no point did the slower-progressing dogs show any significant differences in MSTN levels compared to control dogs.

The TCA cycle (also known as the Krebs or citric acid cycle) was a top PANTHER pathway for fast-progressing dogs at the earlier time point and was among the top PANTHER pathways for slow-progressing dogs at the later time point. Relationships between the TCA cycle and muscular dystrophy/GRMD pathogenesis are not well understood. However, studies indicate a link between muscle wasting (as seen in muscular dystrophies) and dysfunctional energy-related pathways. For example, decreased amounts of adenosine triphosphate (ATP), a product of the TCA cycle, have been observed in dystrophic muscle [74, 75]. This deficit likely arises in part due to increased intracellular calcium [76] and muscle regeneration, resulting in an increased need for ATP production via the TCA cycle [77]. Furthermore, an increase in the activity of TCA cycle enzymes was observed in several brain and muscle tissues of the *mdx* mouse, suggesting that mitochondrial dysfunction and oxidative stress are part of *mdx* pathophysiology [78]. Additional evidence is needed to evaluate whether there is a delay in the onset of oxidative stress and muscle wasting in the slow-progressing dogs compared to fast-progressing dogs.

Inflammation is a typical response to muscle damage, as well as a precursor to the onset of fibrosis. As expected, we identified several immune-related DEGs with functions related specifically to inflammation. Some of these, such as secreted phosphoprotein 1 (SPP1, also known as osteopontin), have been comprehensively described before in the context of DMD, *mdx*, and GRMD pathogenesis and fibrosis [79–82]. SPP1 is strongly up-regulated in DMD and *mdx* [3, 10, 43, 79] as well as GRMD [7, 82] and has been identified as a therapeutic target for DMD [83]. In the current study, we have also confirmed the dramatic up-regulation of SPP1 in GRMD dogs compared to controls (roughly 7-fold increase in expression for both fast- and slow-progressing dogs) using RNA sequencing.

A surprising finding related to inflammation and GRMD was the strong, ~5-6-fold up-regulation of CHI3L1 in fast-progressing vs. control dogs. The connection between CHI3L1 and speed of GRMD disease progression is particularly intriguing. The up-regulation of CHI3L1 has been previously linked with fibrosis in other diseases, most notably idiopathic pulmonary fibrosis [84] and liver fibrosis [85]. CHI3L1 expression in normal human muscle cell culture occurs in a differentiation dependent fashion, being more pronounced in myotubes versus myoblasts [86]. The GRMD response, therefore, likely reflects relatively mature muscle regeneration, as would be expected in the CS muscle by 3 months. The significant increase in CHI3L1 in fast-progressing GRMD dogs could indicate that this gene contributes to accelerated disease progression in GRMD by playing a pro-fibrotic role.

A reciprocal relationship has been demonstrated in myotube cultures between CHI3L1 and the inflammatory cytokine TNFα, with CHI3L1 being induced by and also inhibiting TNFα-induced inflammation [86]. The interplay between TNFα and CHI3L1 is mediated through the NF-κB pathway, which is also involved in DMD inflammation [87]. Germane to disordered metabolism in DMD, CHI3L1 also counteracts TNFα-induced insulin resistance in muscle cells [86]. Therefore, CHI3L1 levels could be increased as part of a feedback mechanism.

Some skeletal muscle gene expression profiles for DMD patients have also shown CHI3L1 to be up-regulated compared to healthy controls [8, 10, 43], and at least one *mdx* expression profile has shown a slight increase in the murine homolog to CHI3L1, *Chil1*, in the skeletal muscle of *mdx* compared to control mice [88]. *Chil1* levels were also slightly increased in the diaphragms of some *mdx* mice compared to control [44]. However, these findings were neither sufficiently remarkable nor consistent to be discussed in any accompanying literature. In fact, some other expression studies found that CHI3L1 expression levels in DMD patients, and *Chil1* levels in *mdx* mice, were not substantially different from those of controls [35, 89–91]. Additional studies are therefore warranted to clarify the nature of the role of CHI3L1 in GRMD inflammation/fibrosis and disease progression and the potential relevance of CHI3L1 in DMD pathogenesis and progression.

The small sample number is a major limitation to this study, but not unusual for a longitudinal study involving a large animal model such as dogs. Furthermore, lack of histological data makes drawing conclusions difficult, as physiological data alone are the basis for categorizing dogs as fast- or slow-progressing without additional evidence for comparing fibrosis levels. The dogs and samples used in this present study have also been used in other studies, in an attempt to retrieve maximal data from a data set that must be as small as reasonably possible. However, given the sample data at hand, this project is particularly valuable in that it provides a number of starting points for future investigations.

## Supporting information

S1 Table Top 10 DEGs with associated log_2_ fold changes and q-values. (XLS)

S2 Table Results for gene ontology and pathway analysis for comparisons across fast, slow, and control dogs. (XLS)

S3 Table List of nonsynonymous SNVs. All identified nonsynonymous SNVs are listed in the worksheet labeled “nonsynonymous”, while those included in top 10 up- and down-regulated DEGs are featured on the worksheet labeled “Included in Top10 lists”.
(XLSX)

## References

[1] Ervasti JM. Dystrophin, its interactions with other proteins, and implications for muscular dystrophy. Biochim Biophys Acta. 2007;1772(2):108–17. doi: 10.1016/j.bbadis.2006.05.010 . 16829057 10.1016/j.bbadis.2006.05.010

[2] Manning J, O'Malley D. What has the mdx mouse model of Duchenne muscular dystrophy contributed to our understanding of this disease? J Muscle Res Cell Motil. 2015;36(2):155–67. doi: 10.1007/s10974-015-9406-4 . 25669899 10.1007/s10974-015-9406-4

[3] Porter JD, Khanna S, Kaminski HJ, Rao JS, Merriam AP, Richmonds CR, et al A chronic inflammatory response dominates the skeletal muscle molecular signature in dystrophin-deficient mdx mice. Hum Mol Genet. 2002;11(3):263–72. . 11823445 10.1093/hmg/11.3.263

[4] Kornegay JN, Bogan JR, Bogan DJ, Childers MK, Grange RW. Golden retriever muscular dystrophy (GRMD): Developing and maintaining a colony and physiological functional measurements. Methods Mol Biol. 2011;709:105–23. Epub 2011/01/05. doi: 10.1007/978-1-61737-982-6_7 . 21194024 10.1007/978-1-61737-982-6_7

[5] Tseng BS, Zhao P, Pattison JS, Gordon SE, Granchelli JA, Madsen RW, et al Regenerated mdx mouse skeletal muscle shows differential mRNA expression. J Appl Physiol (1985). 2002;93(2):537–45. doi: 10.1152/japplphysiol.00202.2002 . 12133862 10.1152/japplphysiol.00202.2002

[6] Galindo CL, Soslow JH, Brinkmeyer-Langford CL, Gupte M, Smith HM, Sengsayadeth S, et al Translating golden retriever muscular dystrophy microarray findings to novel biomarkers for cardiac/skeletal muscle function in Duchenne muscular dystrophy. Pediatr Res. 2016;79(4):629–36. doi: 10.1038/pr.2015.257 ; PubMed Central PMCID: PMCPMC4837049. 26672735 10.1038/pr.2015.257 PMC4837049

[7] Nghiem PP, Hoffman EP, Mittal P, Brown KJ, Schatzberg SJ, Ghimbovschi S, et al Sparing of the dystrophin-deficient cranial sartorius muscle is associated with classical and novel hypertrophy pathways in GRMD dogs. The American journal of pathology. 2013;183(5):1411–24. doi: 10.1016/j.ajpath.2013.07.013 ; PubMed Central PMCID: PMCPMC3814684. 24160322 10.1016/j.ajpath.2013.07.013 PMC3814684

[8] Pescatori M, Broccolini A, Minetti C, Bertini E, Bruno C, D'Amico A, et al Gene expression profiling in the early phases of DMD: a constant molecular signature characterizes DMD muscle from early postnatal life throughout disease progression. FASEB journal: official publication of the Federation of American Societies for Experimental Biology. 2007;21(4):1210–26. doi: 10.1096/fj.06-7285com . 17264171 10.1096/fj.06-7285com

[9] Chen YW, Nagaraju K, Bakay M, McIntyre O, Rawat R, Shi R, et al Early onset of inflammation and later involvement of TGFbeta in Duchenne muscular dystrophy. Neurology. 2005;65(6):826–34. doi: 10.1212/01.wnl.0000173836.09176.c4 . 16093456 10.1212/01.wnl.0000173836.09176.c4

[10] Haslett JN, Sanoudou D, Kho AT, Bennett RR, Greenberg SA, Kohane IS, et al Gene expression comparison of biopsies from Duchenne muscular dystrophy (DMD) and normal skeletal muscle. Proc Natl Acad Sci U S A. 2002;99(23):15000–5. doi: 10.1073/pnas.192571199 ; PubMed Central PMCID: PMCPMC137534. 12415109 10.1073/pnas.192571199 PMC137534

[11] Kornegay JN, Childers MK. Canine inherited dystrophinopathies and centronuclear myopathies In: Childers MK, editor. Regenerative medicine for degenerative muscle diseases. New York: Humana Press; 2016 p. 309–29.

[12] Vieira NM, Elvers I, Alexander MS, Moreira YB, Eran A, Gomes JP, et al Jagged 1 Rescues the Duchenne Muscular Dystrophy Phenotype. Cell. 2015;163(5):1204–13. doi: 10.1016/j.cell.2015.10.049 ; PubMed Central PMCID: PMCPMC4668935. 26582133 10.1016/j.cell.2015.10.049 PMC4668935

[13] Nghiem PP, Bello L, Balog-Alvarez C, Lopez SM, Bettis A, Barnett H, et al Whole genome sequencing reveals a 7 base-pair deletion in DMD exon 42 in a dog with muscular dystrophy. Mammalian genome: official journal of the International Mammalian Genome Society. 2017;28(3–4):106–13. doi: 10.1007/s00335-016-9675-2 ; PubMed Central PMCID: PMCPMC5371640. 28028563 10.1007/s00335-016-9675-2 PMC5371640

[14] Kornegay JN, Bogan JR, Bogan DJ, Childers MK, Grange RW. Golden retriever muscular dystrophy (GRMD): Developing and maintaining a colony and physiological functional measurements In: Duan D, editor. Muscle Gene Therapy: Methods and Protocols. 709 New York: Humana Press; 2011 p. 105–23. 10.1007/978-1-61737-982-6_7 21194024

[15] Kornegay JN, Bogan JR, Bogan DJ, Childers MK, Li J, Nghiem P, et al Canine models of Duchenne muscular dystrophy and their use in therapeutic strategies. Mammalian genome: official journal of the International Mammalian Genome Society. 2012;23(1–2):85–108. Epub 2012/01/06. doi: 10.1007/s00335-011-9382-y . 22218699 10.1007/s00335-011-9382-y PMC3911884

[16] Flanigan KM, Ceco E, Lamar KM, Kaminoh Y, Dunn DM, Mendell JR, et al LTBP4 genotype predicts age of ambulatory loss in duchenne muscular dystrophy. Annals of neurology. 2012 Epub 2013/02/27. doi: 10.1002/ana.23819 . 23440719 10.1002/ana.23819 PMC4106425

[17] Valentine BA, Cooper BJ, de Lahunta A, O'Quinn R, Blue JT. Canine X-linked muscular dystrophy. An animal model of Duchenne muscular dystrophy: clinical studies. Journal of the neurological sciences. 1988;88(1–3):69–81. Epub 1988/12/01. . 3225630 10.1016/0022-510x(88)90206-7

[18] Bartlett RJ, Winand NJ, Secore SL, Singer JT, Fletcher S, Wilton S, et al Mutation segregation and rapid carrier detection of X-linked muscular dystrophy in dogs. American journal of veterinary research. 1996;57(5):650–4. Epub 1996/05/01. . 8723876

[19] Brinkmeyer-Langford C, Balog-Alvarez C, Cai JJ, Davis BW, Kornegay JN. Genome-wide association study to identify potential genetic modifiers in a canine model for Duchenne muscular dystrophy. BMC Genomics. 2016;17:665 doi: 10.1186/s12864-016-2948-z ; PubMed Central PMCID: PMCPMC4994242. 27549615 10.1186/s12864-016-2948-z PMC4994242

[20] Price AL, Zaitlen NA, Reich D, Patterson N. New approaches to population stratification in genome-wide association studies. Nature reviews Genetics. 2010;11(7):459–63. Epub 2010/06/16. doi: 10.1038/nrg2813 ; PubMed Central PMCID: PMC2975875. 20548291 10.1038/nrg2813 PMC2975875

[21] Kornegay JN, Cundiff DD, Bogan DJ, Bogan JR, Okamura CS. The cranial sartorius muscle undergoes true hypertrophy in dogs with golden retriever muscular dystrophy. Neuromuscul Disord. 2003;13(6):493–500. . 12899877 10.1016/s0960-8966(03)00025-7

[22] Rozen S, Skaletsky H. Primer3 on the WWW for general users and for biologist programmers. Methods Mol Biol. 2000;132:365–86. Epub 1999/11/05. . 10547847 10.1385/1-59259-192-2:365

[23] Livak KJ, Schmittgen TD. Analysis of relative gene expression data using real-time quantitative PCR and the 2(-Delta Delta C(T)) Method. Methods. 2001;25(4):402–8. Epub 2002/02/16. doi: 10.1006/meth.2001.1262 . 11846609 10.1006/meth.2001.1262

[24] JMP version 13. Cary, North Carolina, USA: SAS Institute Inc.; 1989–2016.

[25] Kim D, Langmead B, Salzberg SL. HISAT: a fast spliced aligner with low memory requirements. Nat Methods. 2015;12(4):357–60. doi: 10.1038/nmeth.3317 ; PubMed Central PMCID: PMCPMC4655817. 25751142 10.1038/nmeth.3317 PMC4655817

[26] Anders S, Pyl PT, Huber W. HTSeq—a Python framework to work with high-throughput sequencing data. Bioinformatics. 2015;31(2):166–9. doi: 10.1093/bioinformatics/btu638 ; PubMed Central PMCID: PMCPMC4287950. 25260700 10.1093/bioinformatics/btu638 PMC4287950

[27] Love MI, Huber W, Anders S. Moderated estimation of fold change and dispersion for RNA-seq data with DESeq2. Genome Biol. 2014;15(12):550 doi: 10.1186/s13059-014-0550-8 ; PubMed Central PMCID: PMCPMC4302049. 25516281 10.1186/s13059-014-0550-8 PMC4302049

[28] Benjamini Y, Hochberg Y. Controlling the False Discovery Rate: A Practical and Powerful Approach to Multiple Testing. Journal of the Royal Statistical Society, Series B (Methodological). 1995;57(1):289–300.

[29] Mi H, Poudel S, Muruganujan A, Casagrande JT, Thomas PD. PANTHER version 10: expanded protein families and functions, and analysis tools. Nucleic Acids Res. 2016;44(D1):D336–42. doi: 10.1093/nar/gkv1194 ; PubMed Central PMCID: PMCPMC4702852. 26578592 10.1093/nar/gkv1194 PMC4702852

[30] Mi H, Muruganujan A, Casagrande JT, Thomas PD. Large-scale gene function analysis with the PANTHER classification system. Nat Protoc. 2013;8(8):1551–66. doi: 10.1038/nprot.2013.092 . 23868073 10.1038/nprot.2013.092 PMC6519453

[31] Dobin A, Gingeras TR. Mapping RNA-seq Reads with STAR. Curr Protoc Bioinformatics. 2015;51:11 4 1–9. doi: 10.1002/0471250953.bi1114s51 ; PubMed Central PMCID: PMCPMC4631051. 26334920 10.1002/0471250953.bi1114s51 PMC4631051

[32] McKenna A, Hanna M, Banks E, Sivachenko A, Cibulskis K, Kernytsky A, et al The Genome Analysis Toolkit: a MapReduce framework for analyzing next-generation DNA sequencing data. Genome research. 2010;20(9):1297–303. doi: 10.1101/gr.107524.110 ; PubMed Central PMCID: PMCPMC2928508. 20644199 10.1101/gr.107524.110 PMC2928508

[33] DePristo MA, Banks E, Poplin R, Garimella KV, Maguire JR, Hartl C, et al A framework for variation discovery and genotyping using next-generation DNA sequencing data. Nat Genet. 2011;43(5):491–8. doi: 10.1038/ng.806 ; PubMed Central PMCID: PMCPMC3083463. 21478889 10.1038/ng.806 PMC3083463

[34] Van der Auwera GA, Carneiro MO, Hartl C, Poplin R, Del Angel G, Levy-Moonshine A, et al From FastQ data to high confidence variant calls: the Genome Analysis Toolkit best practices pipeline. Curr Protoc Bioinformatics. 2013;43:11 0 1–33. doi: 10.1002/0471250953.bi1110s43 ; PubMed Central PMCID: PMCPMC4243306. 25431634 10.1002/0471250953.bi1110s43 PMC4243306

[35] Porter JD, Merriam AP, Leahy P, Gong B, Khanna S. Dissection of temporal gene expression signatures of affected and spared muscle groups in dystrophin-deficient (mdx) mice. Hum Mol Genet. 2003;12(15):1813–21. . 12874102 10.1093/hmg/ddg197

[36] van Putten M, Kumar D, Hulsker M, Hoogaars WM, Plomp JJ, van Opstal A, et al Comparison of skeletal muscle pathology and motor function of dystrophin and utrophin deficient mouse strains. Neuromuscul Disord. 2012;22(5):406–17. doi: 10.1016/j.nmd.2011.10.011 . 22284942 10.1016/j.nmd.2011.10.011

[37] DiMario JX, Uzman A, Strohman RC. Fiber regeneration is not persistent in dystrophic (MDX) mouse skeletal muscle. Dev Biol. 1991;148(1):314–21. . 1936568 10.1016/0012-1606(91)90340-9

[38] Schiaffino S, Rossi AC, Smerdu V, Leinwand LA, Reggiani C. Developmental myosins: expression patterns and functional significance. Skeletal muscle. 2015;5:22 doi: 10.1186/s13395-015-0046-6 ; PubMed Central PMCID: PMCPMC4502549. 26180627 10.1186/s13395-015-0046-6 PMC4502549

[39] Lyons GE, Ontell M, Cox R, Sassoon D, Buckingham M. The expression of myosin genes in developing skeletal muscle in the mouse embryo. The Journal of cell biology. 1990;111(4):1465–76. ; PubMed Central PMCID: PMCPMC2116224. 2211821 10.1083/jcb.111.4.1465 PMC2116224

[40] Bakay M, Zhao P, Chen J, Hoffman EP. A web-accessible complete transcriptome of normal human and DMD muscle. Neuromuscul Disord. 2002;12 Suppl 1:S125–41. . 12206807 10.1016/s0960-8966(02)00093-7

[41] Noguchi S, Tsukahara T, Fujita M, Kurokawa R, Tachikawa M, Toda T, et al cDNA microarray analysis of individual Duchenne muscular dystrophy patients. Hum Mol Genet. 2003;12(6):595–600. . 12620965

[42] Wang Q, Reiter RS, Huang QQ, Jin JP, Lin JJ. Comparative studies on the expression patterns of three troponin T genes during mouse development. Anat Rec. 2001;263(1):72–84. . 11331973 10.1002/ar.1078

[43] Chen YW, Zhao P, Borup R, Hoffman EP. Expression profiling in the muscular dystrophies: identification of novel aspects of molecular pathophysiology. The Journal of cell biology. 2000;151(6):1321–36. ; PubMed Central PMCID: PMCPMC2190600. 11121445 10.1083/jcb.151.6.1321 PMC2190600

[44] Porter JD, Merriam AP, Leahy P, Gong B, Feuerman J, Cheng G, et al Temporal gene expression profiling of dystrophin-deficient (mdx) mouse diaphragm identifies conserved and muscle group-specific mechanisms in the pathogenesis of muscular dystrophy. Hum Mol Genet. 2004;13(3):257–69. doi: 10.1093/hmg/ddh033 . 14681298 10.1093/hmg/ddh033

[45] Christian SL, Brune CW, Sudi J, Kumar RA, Liu S, Karamohamed S, et al Novel submicroscopic chromosomal abnormalities detected in autism spectrum disorder. Biol Psychiatry. 2008;63(12):1111–7. doi: 10.1016/j.biopsych.2008.01.009 ; PubMed Central PMCID: PMCPMC2440346. 18374305 10.1016/j.biopsych.2008.01.009 PMC2440346

[46] Wei X, Walia V, Lin JC, Teer JK, Prickett TD, Gartner J, et al Exome sequencing identifies GRIN2A as frequently mutated in melanoma. Nat Genet. 2011;43(5):442–6. doi: 10.1038/ng.810 ; PubMed Central PMCID: PMCPMC3161250. 21499247 10.1038/ng.810 PMC3161250

[47] Wittkowski KM, Sonakya V, Bigio B, Tonn MK, Shic F, Ascano M, et al A novel computational biostatistics approach implies impaired dephosphorylation of growth factor receptors as associated with severity of autism. Transl Psychiatry. 2014;4:e354 doi: 10.1038/tp.2013.124 ; PubMed Central PMCID: PMCPMC3905234. 24473445 10.1038/tp.2013.124 PMC3905234

[48] Wu JY, Kuban KC, Allred E, Shapiro F, Darras BT. Association of Duchenne muscular dystrophy with autism spectrum disorder. J Child Neurol. 2005;20(10):790–5. doi: 10.1177/08830738050200100201 . 16417872 10.1177/08830738050200100201

[49] Ameye L, Young MF. Mice deficient in small leucine-rich proteoglycans: novel in vivo models for osteoporosis, osteoarthritis, Ehlers-Danlos syndrome, muscular dystrophy, and corneal diseases. Glycobiology. 2002;12(9):107R–16R. . 12213783 10.1093/glycob/cwf065

[50] Hoffmann H, Schiene-Fischer C. Functional aspects of extracellular cyclophilins. Biol Chem. 2014;395(7–8):721–35. doi: 10.1515/hsz-2014-0125 . 24713575 10.1515/hsz-2014-0125

[51] Yao Q, Li M, Yang H, Chai H, Fisher W, Chen C. Roles of cyclophilins in cancers and other organ systems. World J Surg. 2005;29(3):276–80. doi: 10.1007/s00268-004-7812-7 . 15706440 10.1007/s00268-004-7812-7

[52] Gazzerro E, Baldassari S, Assereto S, Fruscione F, Pistorio A, Panicucci C, et al Enhancement of Muscle T Regulatory Cells and Improvement of Muscular Dystrophic Process in mdx Mice by Blockade of Extracellular ATP/P2X Axis. The American journal of pathology. 2015;185(12):3349–60. doi: 10.1016/j.ajpath.2015.08.010 . 26465071 10.1016/j.ajpath.2015.08.010

[53] Villalta SA, Rosenthal W, Martinez L, Kaur A, Sparwasser T, Tidball JG, et al Regulatory T cells suppress muscle inflammation and injury in muscular dystrophy. Science translational medicine. 2014;6(258):258ra142 doi: 10.1126/scitranslmed.3009925 ; PubMed Central PMCID: PMCPMC4889432. 25320234 10.1126/scitranslmed.3009925 PMC4889432

[54] Burzyn D, Kuswanto W, Kolodin D, Shadrach JL, Cerletti M, Jang Y, et al A special population of regulatory T cells potentiates muscle repair. Cell. 2013;155(6):1282–95. doi: 10.1016/j.cell.2013.10.054 ; PubMed Central PMCID: PMCPMC3894749. 24315098 10.1016/j.cell.2013.10.054 PMC3894749

[55] Wuebbles RD, Sarathy A, Kornegay JN, Burkin DJ. Levels of alpha7 integrin and laminin-alpha2 are increased following prednisone treatment in the mdx mouse and GRMD dog models of Duchenne muscular dystrophy. Disease models & mechanisms. 2013;6(5):1175–84. doi: 10.1242/dmm.012211 ; PubMed Central PMCID: PMCPMC3759337. 23846963 10.1242/dmm.012211 PMC3759337

[56] Giancotti FG, Ruoslahti E. Integrin signaling. Science. 1999;285(5430):1028–32. . 10446041 10.1126/science.285.5430.1028

[57] Rozo M, Li L, Fan CM. Targeting beta1-integrin signaling enhances regeneration in aged and dystrophic muscle in mice. Nature medicine. 2016;22(8):889–96. doi: 10.1038/nm.4116 ; PubMed Central PMCID: PMCPMC4974124. 27376575 10.1038/nm.4116 PMC4974124

[58] Valentine BA, Cooper BJ. Canine X-linked muscular dystrophy: selective involvement of muscles in neonatal dogs. Neuromuscul Disord. 1991;1(1):31–8. . 1840414 10.1016/0960-8966(91)90040-y

[59] Nguyen F, Cherel Y, Guigand L, Goubault-Leroux I, Wyers M. Muscle lesions associated with dystrophin deficiency in neonatal golden retriever puppies. J Comp Pathol. 2002;126(2–3):100–8. doi: 10.1053/jcpa.2001.0526 . 11944998 10.1053/jcpa.2001.0526

[60] Zhao Y, Haginoya K, Sun G, Dai H, Onuma A, Iinuma K. Platelet-derived growth factor and its receptors are related to the progression of human muscular dystrophy: an immunohistochemical study. The Journal of pathology. 2003;201(1):149–59. doi: 10.1002/path.1414 . 12950028 10.1002/path.1414

[61] Tidball JG, Spencer MJ, St Pierre BA. PDGF-receptor concentration is elevated in regenerative muscle fibers in dystrophin-deficient muscle. Exp Cell Res. 1992;203(1):141–9. . 1426037 10.1016/0014-4827(92)90049-e

[62] Uezumi A, Ito T, Morikawa D, Shimizu N, Yoneda T, Segawa M, et al Fibrosis and adipogenesis originate from a common mesenchymal progenitor in skeletal muscle. J Cell Sci. 2011;124(Pt 21):3654–64. doi: 10.1242/jcs.086629 . 22045730 10.1242/jcs.086629

[63] Uezumi A, Fukada S, Yamamoto N, Takeda S, Tsuchida K. Mesenchymal progenitors distinct from satellite cells contribute to ectopic fat cell formation in skeletal muscle. Nat Cell Biol. 2010;12(2):143–52. doi: 10.1038/ncb2014 . 20081842 10.1038/ncb2014

[64] Ito T, Ogawa R, Uezumi A, Ohtani T, Watanabe Y, Tsujikawa K, et al Imatinib attenuates severe mouse dystrophy and inhibits proliferation and fibrosis-marker expression in muscle mesenchymal progenitors. Neuromuscul Disord. 2013;23(4):349–56. doi: 10.1016/j.nmd.2012.10.025 . 23313020 10.1016/j.nmd.2012.10.025

[65] Sandri M. Signaling in muscle atrophy and hypertrophy. Physiology (Bethesda). 2008;23:160–70. doi: 10.1152/physiol.00041.2007 . 18556469 10.1152/physiol.00041.2007

[66] Sakuma K, Aoi W, Yamaguchi A. The intriguing regulators of muscle mass in sarcopenia and muscular dystrophy. Front Aging Neurosci. 2014;6:230 doi: 10.3389/fnagi.2014.00230 ; PubMed Central PMCID: PMCPMC4148637. 25221510 10.3389/fnagi.2014.00230 PMC4148637

[67] McPherron AC, Lawler AM, Lee SJ. Regulation of skeletal muscle mass in mice by a new TGF-beta superfamily member. Nature. 1997;387(6628):83–90. doi: 10.1038/387083a0 . 9139826 10.1038/387083a0

[68] McPherron AC, Lee SJ. Double muscling in cattle due to mutations in the myostatin gene. Proc Natl Acad Sci U S A. 1997;94(23):12457–61. ; PubMed Central PMCID: PMCPMC24998. 9356471 10.1073/pnas.94.23.12457 PMC24998

[69] Wagner KR, Liu X, Chang X, Allen RE. Muscle regeneration in the prolonged absence of myostatin. Proc Natl Acad Sci U S A. 2005;102(7):2519–24. doi: 10.1073/pnas.0408729102 ; PubMed Central PMCID: PMCPMC548322. 15699335 10.1073/pnas.0408729102 PMC548322

[70] Bogdanovich S, Krag TO, Barton ER, Morris LD, Whittemore LA, Ahima RS, et al Functional improvement of dystrophic muscle by myostatin blockade. Nature. 2002;420(6914):418–21. doi: 10.1038/nature01154 . 12459784 10.1038/nature01154

[71] Wagner KR, McPherron AC, Winik N, Lee SJ. Loss of myostatin attenuates severity of muscular dystrophy in mdx mice. Ann Neurol. 2002;52(6):832–6. doi: 10.1002/ana.10385 . 12447939 10.1002/ana.10385

[72] Qiao C, Li J, Zheng H, Bogan J, Li J, Yuan Z, et al Hydrodynamic limb vein injection of adeno-associated virus serotype 8 vector carrying canine myostatin propeptide gene into normal dogs enhances muscle growth. Hum Gene Ther. 2009;20(1):1–10. doi: 10.1089/hum.2008.135 ; PubMed Central PMCID: PMCPMC2855246. 18828709 10.1089/hum.2008.135 PMC2855246

[73] Bish LT, Sleeper MM, Forbes SC, Morine KJ, Reynolds C, Singletary GE, et al Long-term systemic myostatin inhibition via liver-targeted gene transfer in golden retriever muscular dystrophy. Hum Gene Ther. 2011;22(12):1499–509. doi: 10.1089/hum.2011.102 ; PubMed Central PMCID: PMCPMC3237695. 21787232 10.1089/hum.2011.102 PMC3237695

[74] Austin L, de Niese M, McGregor A, Arthur H, Gurusinghe A, Gould MK. Potential oxyradical damage and energy status in individual muscle fibres from degenerating muscle diseases. Neuromuscul Disord. 1992;2(1):27–33. . 1525555 10.1016/0960-8966(92)90023-y

[75] Cole MA, Rafael JA, Taylor DJ, Lodi R, Davies KE, Styles P. A quantitative study of bioenergetics in skeletal muscle lacking utrophin and dystrophin. Neuromuscul Disord. 2002;12(3):247–57. . 11801396 10.1016/s0960-8966(01)00278-4

[76] Dunn JF, Radda GK. Total ion content of skeletal and cardiac muscle in the mdx mouse dystrophy: Ca2+ is elevated at all ages. Journal of the neurological sciences. 1991;103(2):226–31. . 1880541 10.1016/0022-510x(91)90168-7

[77] Rybalka E, Timpani CA, Cooke MB, Williams AD, Hayes A. Defects in mitochondrial ATP synthesis in dystrophin-deficient mdx skeletal muscles may be caused by complex I insufficiency. PLoS One. 2014;9(12):e115763 doi: 10.1371/journal.pone.0115763 ; PubMed Central PMCID: PMCPMC4277356. 25541951 10.1371/journal.pone.0115763 PMC4277356

[78] Comim CM, Hoepers A, Ventura L, Freiberger V, Dominguini D, Mina F, et al Activity of Krebs cycle enzymes in mdx mice. Muscle Nerve. 2016;53(1):91–5. doi: 10.1002/mus.24704 . 25965940 10.1002/mus.24704

[79] Zanotti S, Gibertini S, Di Blasi C, Cappelletti C, Bernasconi P, Mantegazza R, et al Osteopontin is highly expressed in severely dystrophic muscle and seems to play a role in muscle regeneration and fibrosis. Histopathology. 2011;59(6):1215–28. doi: 10.1111/j.1365-2559.2011.04051.x . 22175901 10.1111/j.1365-2559.2011.04051.x

[80] Pardo A, Gibson K, Cisneros J, Richards TJ, Yang Y, Becerril C, et al Up-regulation and profibrotic role of osteopontin in human idiopathic pulmonary fibrosis. PLoS Med. 2005;2(9):e251 doi: 10.1371/journal.pmed.0020251 ; PubMed Central PMCID: PMCPMC1198037. 16128620 10.1371/journal.pmed.0020251 PMC1198037

[81] Vetrone SA, Montecino-Rodriguez E, Kudryashova E, Kramerova I, Hoffman EP, Liu SD, et al Osteopontin promotes fibrosis in dystrophic mouse muscle by modulating immune cell subsets and intramuscular TGF-beta. J Clin Invest. 2009;119(6):1583–94. doi: 10.1172/JCI37662 ; PubMed Central PMCID: PMCPMC2689112. 19451692 10.1172/JCI37662 PMC2689112

[82] Kornegay JN, Spurney CF, Nghiem PP, Brinkmeyer-Langford CL, Hoffman EP, Nagaraju K. Pharmacologic management of Duchenne muscular dystrophy: target identification and preclinical trials. ILAR J. 2014;55(1):119–49. doi: 10.1093/ilar/ilu011 ; PubMed Central PMCID: PMCPMC4158345. 24936034 10.1093/ilar/ilu011 PMC4158345

[83] Capote J, Kramerova I, Martinez L, Vetrone S, Barton ER, Sweeney HL, et al Osteopontin ablation ameliorates muscular dystrophy by shifting macrophages to a pro-regenerative phenotype. The Journal of cell biology. 2016;213(2):275–88. doi: 10.1083/jcb.201510086 ; PubMed Central PMCID: PMCPMC5084275. 27091452 10.1083/jcb.201510086 PMC5084275

[84] Zhou Y, Peng H, Sun H, Peng X, Tang C, Gan Y, et al Chitinase 3-like 1 suppresses injury and promotes fibroproliferative responses in Mammalian lung fibrosis. Science translational medicine. 2014;6(240):240ra76 doi: 10.1126/scitranslmed.3007096 ; PubMed Central PMCID: PMCPMC4340473. 24920662 10.1126/scitranslmed.3007096 PMC4340473

[85] Berres ML, Papen S, Pauels K, Schmitz P, Zaldivar MM, Hellerbrand C, et al A functional variation in CHI3L1 is associated with severity of liver fibrosis and YKL-40 serum levels in chronic hepatitis C infection. J Hepatol. 2009;50(2):370–6. doi: 10.1016/j.jhep.2008.09.016 . 19070929 10.1016/j.jhep.2008.09.016

[86] Gorgens SW, Eckardt K, Elsen M, Tennagels N, Eckel J. Chitinase-3-like protein 1 protects skeletal muscle from TNFalpha-induced inflammation and insulin resistance. Biochem J. 2014;459(3):479–88. doi: 10.1042/BJ20131151 . 24512683 10.1042/BJ20131151

[87] Monici MC, Aguennouz M, Mazzeo A, Messina C, Vita G. Activation of nuclear factor-kappaB in inflammatory myopathies and Duchenne muscular dystrophy. Neurology. 2003;60(6):993–7. . 12654966 10.1212/01.wnl.0000049913.27181.51

[88] Chandrasekharan K, Yoon JH, Xu Y, deVries S, Camboni M, Janssen PM, et al A human-specific deletion in mouse Cmah increases disease severity in the mdx model of Duchenne muscular dystrophy. Science translational medicine. 2010;2(42):42ra54 doi: 10.1126/scitranslmed.3000692 ; PubMed Central PMCID: PMCPMC2950110. 20668298 10.1126/scitranslmed.3000692 PMC2950110

[89] Dadgar S, Wang Z, Johnston H, Kesari A, Nagaraju K, Chen YW, et al Asynchronous remodeling is a driver of failed regeneration in Duchenne muscular dystrophy. The Journal of cell biology. 2014;207(1):139–58. doi: 10.1083/jcb.201402079 ; PubMed Central PMCID: PMCPMC4195829. 25313409 10.1083/jcb.201402079 PMC4195829

[90] Bakay M, Wang Z, Melcon G, Schiltz L, Xuan J, Zhao P, et al Nuclear envelope dystrophies show a transcriptional fingerprint suggesting disruption of Rb-MyoD pathways in muscle regeneration. Brain. 2006;129(Pt 4):996–1013. doi: 10.1093/brain/awl023 . 16478798 10.1093/brain/awl023

[91] Porter JD, Merriam AP, Khanna S, Andrade FH, Richmonds CR, Leahy P, et al Constitutive properties, not molecular adaptations, mediate extraocular muscle sparing in dystrophic mdx mice. FASEB journal: official publication of the Federation of American Societies for Experimental Biology. 2003;17(8):893–5. doi: 10.1096/fj.02-0810fje . 12670877 10.1096/fj.02-0810fje

