# Correction: Expression profiling of disease progression in canine model of Duchenne muscular dystrophy

**DOI:** 10.1371/journal.pone.0236916

**Published:** 2020-07-23

**Authors:** Candice Brinkmeyer-Langford, Candice Chu, Cynthia Balog-Alvarez, Xue Yu, James J. Cai, Mary Nabity, Joe N. Kornegay

Fig 2 is missing panel C. Please see the complete, correct Fig 2 here.

**Fig 2:**
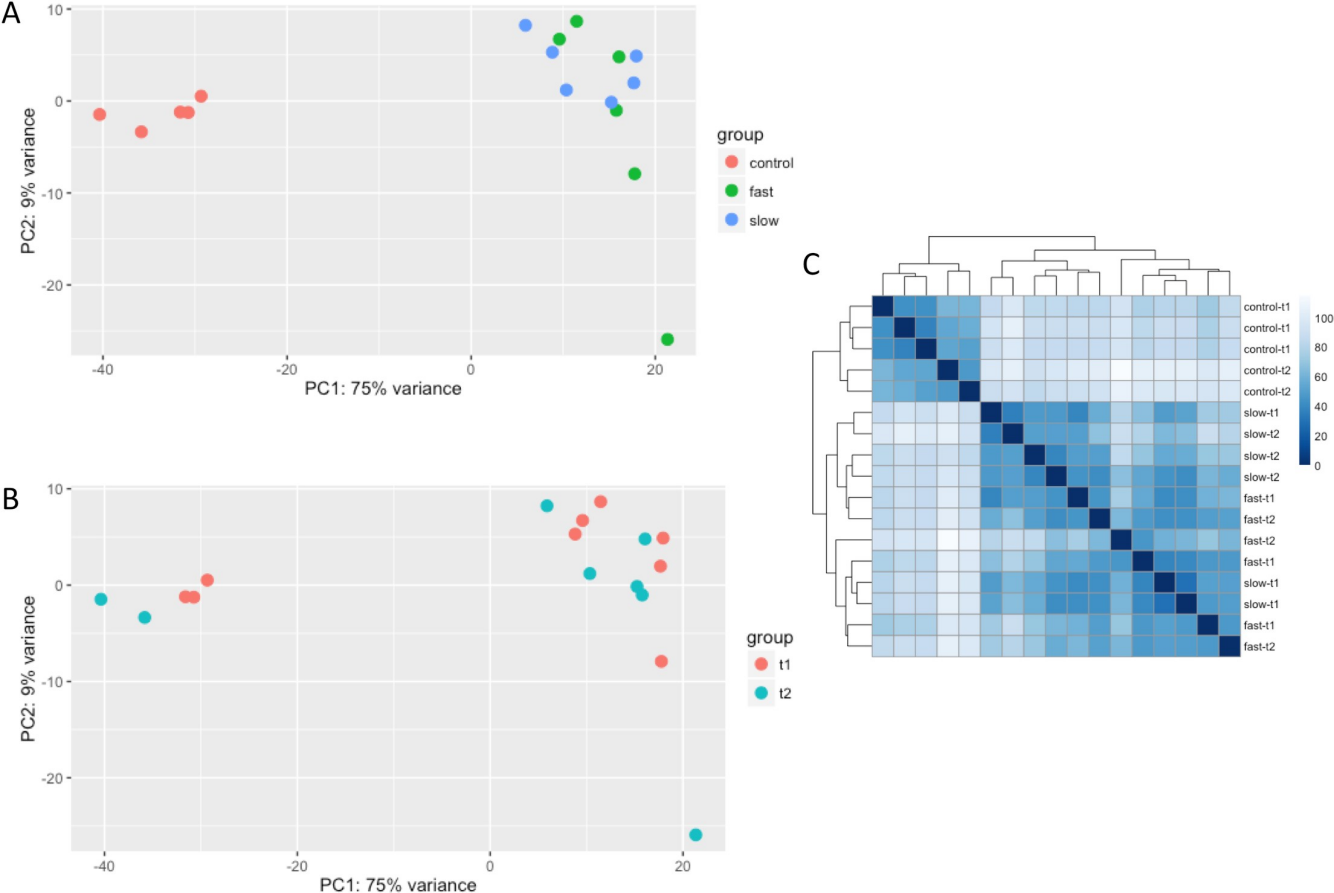
Principal component and hierarchical analysis for all dogs at both time points. Principal component 1 (PC1) and Principal component 2 (PC2) were identified by logarithm transformation in DESeq2 at two time points. 75% and 9% variance were explained by PC1 and PC2, respectively. A) shows the principal component analysis for the three groups of dogs: red circles indicate controls, green represents fast-progressing dogs, and blue represents slower-progressing dogs. B) shows the principal component analysis for the two time points: here, red circles represent T1 (age 3 months), and green circles represent T2 (age 6 months). C) is a heatmap showing sample-to-sample distances. Distance was analyzed by logarithm transformation in DESeq2.
